# Fisher's combined p-value for detecting differentially expressed genes using Affymetrix expression arrays

**DOI:** 10.1186/1471-2164-8-96

**Published:** 2007-04-09

**Authors:** Ann Hess, Hari Iyer

**Affiliations:** 1 Department of Statistics, Colorado State University, Fort, Collins, CO, 80523, USA

## Abstract

**Background:**

Currently, most tests of differential gene expression using Affymetrix expression array data are performed using expression summary values representing each probe set on a microarray. Recently testing methods have been proposed which incorporate probe level information. We propose a new approach that uses Fisher's method of combining evidence from multiple sources of information. Specifically, we combine p-values from probe level tests of significance.

**Results:**

The combined p method and other competing methods were compared using three spike-in datasets (where probe sets corresponding differentially spiked transcripts are known) and array data from a biological study validated with qRT-PCR. Based on power and false discovery rates computed for the spike-in datasets, we demonstrate that the combined p method compares favorably with other methods. We find that probe level testing methods select many of the same genes as differentially expressed. We illustrate the use of the combined p method for diagnostic purposes using examples.

**Conclusion:**

Combined p is a promising alternative to existing methods of testing for differential gene expression. In addition, the combined p method is particularly well suited as a diagnostic tool for exploratory analysis of microarray data.

## Background

Microarrays allow researchers to examine the expression of thousands of genes simultaneously. Affymetrix arrays use groups of oligonucleotide probes, called probe sets, to represent genes of interest on an array. The primary goal of many experiments using Affymetrix expression arrays is to identify a group of genes that is differentially expressed between two or more conditions.

When microarray experiments first started gaining popularity, a simple 2 fold cutoff was used as a threshold to identify differentially expressed genes. Currently, a common approach to identifying differentially expressed genes is to calculate an expression index for each probe set and array and use these expression indices as the basis for statistical testing. In other words, the probe level information is combined into a summary value by probe set and array, then this summary information is used to test for differential expression. The most popular methods for calculating expression indices include Affymetrix Microarray Suite 5 (MAS5) [1], model based expression index (MBEI) [2] and robust multi-chip average (RMA) [3]. Examples of statistical tests to identify differentially expressed genes based on expression indices include the t-test, the non-parametric Wilcoxon rank sum test, Bayesian methods [4-6] and permutation based methods [7].

Recently a number of authors have presented testing methods based on probe values. Individual probes contain information about the abundance of a particular transcript. A two-way ANOVA model of probe values can be used to test for differential gene expression [8]. The ChipStat algorithm tests for differential gene expression using probe level comparisons [9]. The median t-statistic from all probes in a probe set has been used as a test statistic for differential expression of the whole probe set [10]. The S-score was developed for detecting differentially expressed genes based on PM-MM differences without replication [11]. The PPLR (probability of positive log-ratio) method uses information about probe level variability [12]

Clearly there are many methods available for analyzing data resulting from microarray experiments. Furthermore, different methods generally lead to different groups of genes identified as differentially expressed. The availability of spike-in datasets makes it possible to compare methods in a setting where the truth is known. A "good" method will have high power to detect differentially expressed genes but low false discovery rate (FDR). The false discovery rate is defined as the ratio of the expected number of falsely rejected hypotheses to the total number of rejected hypotheses [13]. For microarrays, where thousands of genes are being tested simultaneously, it is reasonable to focus on FDR instead of false positive rate (FPR).  It has also been noted that when identifying differentially expressed genes is the primary objective of the experiment, the aim of the corresponding analysis should be to rank the genes in order of evidence of differential expression [4]. The focus on power, FDR and rank have lead many investigators to rely on receiver operator characteristic (ROC) curves [8,10,14,15] and FDR plots [4,8] when comparing methods for detecting differentially expressed genes. Diagnostics can be employed to identify outlying probe sets or to understand why a gene of interest was not identified as differentially expressed.

We propose using Fisher's combined p method [16] to combine probe level tests of differential expression. Using three spike-in datasets and array data from a biological study, we compare the combined p method to the ANOVA method [8], Cyber-T [6], median t method [10], moderated t-test [4] and the original t-test. In addition to a comparison of methods, some suggested diagnostics based on probe level tests are also presented.

## Results

In order to compare the performance of the methods, we use three different spike-in datasets (where probe sets corresponding to spiked-in transcripts are known) and array data from a biological study validated with qRT-PCR. The focus of this paper is on detecting differentially expressed genes, so the background correction and normalization methods are kept constant for each of the datasets considered. High power to detect differentially expressed genes and low false discovery rate (FDR) are desirable. With these measures in mind we consider ROC curves and FDR plots for each of the methods and datasets. All rankings are based on p-values. The rankings are calculated across comparisons for each dataset. We also use selection curves to examine what genes are selected in common between combined p and other methods. All programming was done in R [17] using Bioconductor [18].

### Data used for Comparison

The "Golden Spike" data employs the Affymetrix DrosGenome1 GeneChip [14]. The DrosGenome1 GeneChip has a total of 14,010 probe sets, typically with 14 probe pairs. Three control arrays and three spike-in arrays were used. A total of 1,331 probe sets have an increased concentration between the control and spike-in samples, 2,535 probe sets have equal concentration and the remaining 10,144 probe sets were empty on both the control and spike-in arrays. For the 1,331 true positives, the *log*_2 _fold changes range from 0.26 to 2. All methods were applied to MAS background corrected and probe level loess subset normalized data, since this combination performed best in the original comparison. Tests based on probe values (ANOVA, combined p and median t) were carried out on the background corrected, normalized and *log*_2 _transformed PM-only values. Expression indices were computed using Tukey biweight average (summary.method = "mas" using the expresso command in Bioconductor) applied to background corrected and normalized PM-only values. Tests based on expression indices (Cyber-T, moderated t and ordinary t) were carried out on the *log*_2 _transformed expression indices. All methods except the two-way ANOVA method and Cyber-T used t-tests assuming equal variance.

The Gene Logic spike-in tonsil data employs the Affymetrix HG-U95A GeneChip [10]. The HG-U95A GeneChip has a total of 12,626 probe sets, typically with 16 probe pairs. This data consists of 3 technical replicates of 12 different hybridization mixtures each with 11 spiked cRNA transcripts. The spiked transcript concentrations range from 0.5 pM to 100 pM. There are 66 pairwise comparisons with 11 true positives per comparison for a total of 726 true positives with *log*_2 _fold changes between -7.64 and +7.64. Tests based on probe values were applied to RMA background corrected, quantiles normalized data and *log*_2 _transformed PM-only values. Tests based on expression indices were carried out on the RMA probe set summary values. The RMA probe set summary algorithm uses only PM values and employs RMA background correction and quantiles normalization. RMA expression indices are reported on the *log*_2 _scale. All methods were based on fitting an ANOVA model and then using contrasts to estimate and test pairwise "treatment" differences.

The Affymetrix Latin Square data is based on the Affymetrix HG-U133A GeneChip [19]. The HG-U133A GeneChip has 22,300 probe sets, typically with 11 probe pairs. This dataset consists of 3 technical replicates of 14 separate hybridizations with 42 spiked transcripts in a complex human background. The spiked transcript concentrations range from 0.125 pM to 512 pM. There are 91 pairwise comparisons with 42 true positives per comparison for a total of 3822 true positives with *log*_2 _fold changes between -12 and +12. Due to concerns about cross-hybridization, 145 probe sets suspected of cross hybridizing with the spike-in transcripts were removed from the analysis. The list of likely cross hybridizing probe sets was obtained from the *affycomp *package from Bioconductor [20]. According to the *affycomp *help file, "The sequences of each spiked-in clone were collected and blasted against all HG-U133A target sequences. Target sequences are the 600 bp regions from which probes were selected. Thresholds of 100, 150 and 200 bp were used." We used a 200 bp threshold for removal. Tests based on probe values were applied to RMA background corrected, quantiles normalized data and *log*_2 _transformed PM-only values. Tests based on expression indices were carried out on the RMA probe set summary values. All methods were based on fitting an ANOVA model and then using contrasts to estimate and test pairwise "treatment" differences.

Finally, we consider the MCAT data from Qin *et al. *[21] which includes data from Affymetrix expression arrays as well as corresponding qRT-PCR results. RNA samples were collected from heart tissue from 24 mice in an unbalanced 2 × 2 factorial design. The 24 mice were young or old, wild-type or carried the MCAT transgene. There were 6 young wild-type (YWT) mice, 8 young MCAT (YMCAT) mice, 5 old wild-type (OWT) mice and 5 old MCAT (OMCAT) mice. Twenty four Affymetrix MG-U74av2 GeneChips were employed. The MG-U74av2 GeneChip has 12,488 probe sets, typically with 16 probe pairs. Quantitative RT-PCR measurements from 47 genes were taken on the same 24 RNA samples. qRT-PCR is often considered a "gold-standard" method of measuring gene expression. We note that the 47 genes assayed with qRT-PCR were not randomly selected, but selected based on "primer availability, initial evidence of differential expression, signal intensity, and biological interest" [21]. We consider three comparisons: YMCAT versus YWT, OMCAT versus OWT and YWT versus OWT. Tests based on probe values were applied to RMA background corrected, quantiles normalized data and *log*_2 _transformed PM-only values. Tests based on expression indices were carried out on the RMA probe set summary values. All methods except the two-way ANOVA method and Cyber-T used a t-test assuming equal variance.

### ROC curves

**Figure 1:**
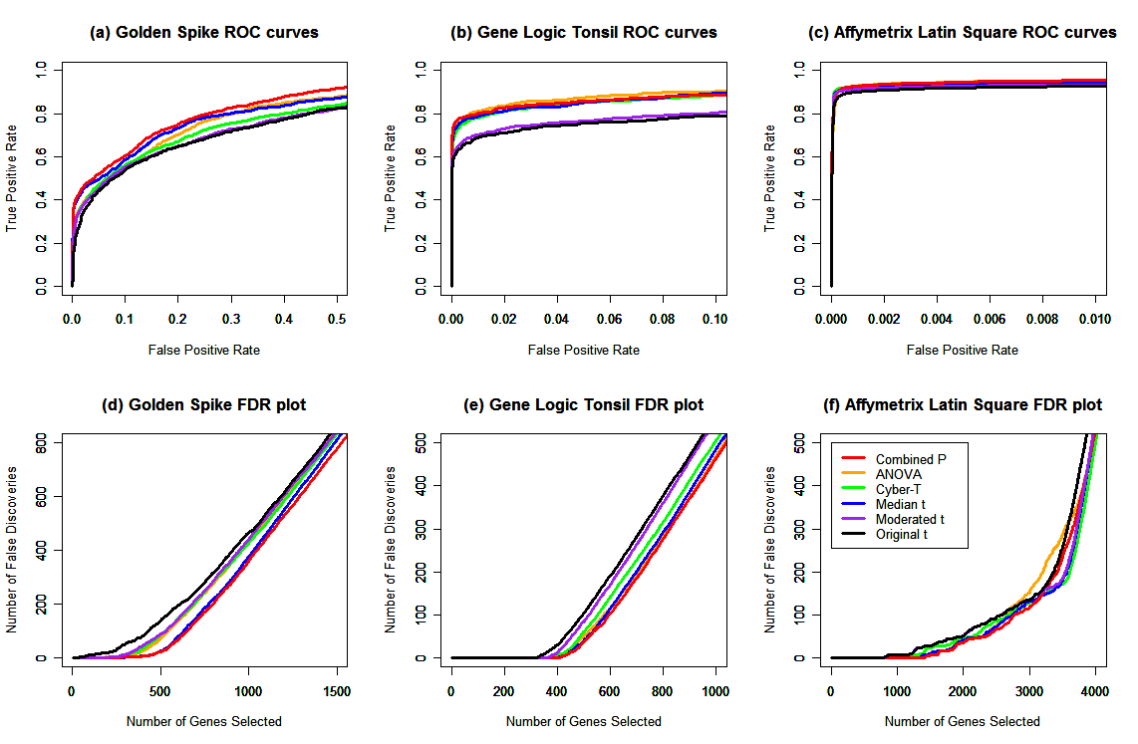
**ROC and FDR plots for each of the spike-in datasets**. (a) ROC curves for the Golden Spike data. (b) ROC curves for the Gene Logic Tonsil data. (c) ROC curves for the Affymetrix Latin Square data. (d) FDR plot for the Golden Spike data. (e) FDR plot for the Gene Logic Tonsil data. (f) FDR plot for the Affymetrix Latin Square data.

The ROC curves show the true positive rate, or power, plotted against the false positive rate (FPR). The ROC curves for the each of the spike-in datasets are shown in Figure 1(a–c). The ideal situation with full power and no false positives corresponds to the upper left corner of the plot. We do not show the full range of false positive rates as we feel that this would be misleading. For example, for the Gene Logic Tonsil data, a false *positive *rate of 0.01 with full power would correspond to a false *discovery *rate of 0.92!

The ROC curves show that the performance of each of the methods is dependent upon the dataset. All methods perform best on the Affymetrix Latin Square data. However, we are primarily concerned with the relative performance of the methods.

### FDR plots

Curves depicting the false discovery rates for the different gene selection statistics for the each of the spike-in datasets are shown in Figure 1(d–f). These curves indicate the number of false discoveries when a given number of top ranked genes is selected as differentially expressed. This graph is a useful comparison representing the scenario where the investigator is primarily interested in ranking genes and choosing a number of the top ranked genes for further follow up and verification, typically using RT-PCR.

The FDR plots focus on the performance of the methods when the FDR is reasonably small. This is the range of practical interest. All methods perform best for the Affymetrix Latin Square data, but the relative performance is of primary concern.

### Ranks of Differentially Spiked Transcripts

The interquartile range (IQR) of ranks by method for known differentially spiked transcripts are shown in Table 1. The ideal ranking where all true positives are ranked above any false positives is also shown for each of the datasets. The rankings are based on the calculated p-values for each of the methods. We expect differentially spiked transcripts to have small p-values and be ranked high. For the Gene Logic Tonsil and Affymetrix Latin Square data, for which we consider multiple pairwise comparisons, we rank the p-values from all comparisons together. Although it is possible to rank the p-values from each comparison separately, we feel that ranking the comparisons together reflects realistic experimental protocol. The combined p method comes closest to the ideal ranking for all three spike-in datasets.

### Power over the Range of Intensity

In order to examine the power of each of the methods over the range of intensity values, the intensity of each of the true positives was calculated as the average of the *log*_2_(*PM*) values. The power was calculated as the proportion of the true positives that was detected while maintaining an overall false discovery rate less than or equal to 0.05. The power for each of the four intensity quartiles as well as overall power for each of the datasets and methods is shown in Table 2.

For the Golden Spike and Gene Logic datasets, combined p yields the highest power overall and for each of the intensity quartiles. For the Affymetrix Latin Square data, Cyber-T yields the highest power overall and for each of the intensity quartiles. We note that the power is calculated at a specified false discovery rate and that relative performance of the methods might vary based on the chosen value of the false discovery rate.

### Observed False Positive Rates

The observed false positive rates for each of the methods when a raw p-value cutoff of 0.01 is used to identify differentially spiked transcripts are shown in Table 3. Because the combined p method uses the minimum of the two one-sided p-values, we use a p-value cutoff of 0.005 for the combined p method only. If we are interested in determining whether a specific gene is differentially expressed, the raw p-values instead of multiple testing adjusted values are appropriate and the comparisonwise error rate is of interest.

In order to better control the false positive rate and to adjust for variability between methods, we propose calibrating the p-values. Specifically, our calibration set is comprised of those probe sets called "Absent" on all arrays according to the MAS Absent/Present call algorithm (computed in Bioconductor). Since transcripts for these probe sets do not appear to be present above background, it seems reasonable to assume that they are not differentially expressed. A calibrated p-value of 0.01 corresponds to the first percentile of the p-values from the calibration set. For the Golden Spike data, 66% of probe sets were called Absent on all six arrays. For the Gene Logic Tonsil data, 25% of probe sets were called Absent on all 36 arrays. For the Affymetrix Latin Square data, 39% of probe sets were called Absent on all 42 arrays. The observed false positive rates for each of the methods when a calibrated p-value cutoff of 0.01 is used to identify differentially expressed genes are shown in Table 3. A p-value cutoff of 0.005 was again used for the combined p method.

None of the methods maintain a false positive rate close to the stated *α *level of 0.01 when using raw p-values. Using a calibrated p-value reduces the false positive rates for all methods except median t.

### Selection Curves

**Figure 2:**
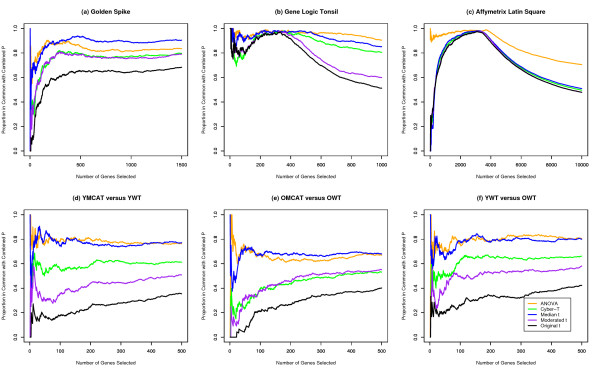
**Selection Curves**. The selection curves show the proportion of genes selected in common with each of the other methods when a given number of top ranked genes is selected as differentially expressed by combined p. (a) Selection curves for the Golden Spike data. (b) Selection curves for the Gene Logic Tonsil data. (c) Selection curves for the Affymetrix Latin Square data. (d) Selection curves for the YMCAT versus YWT comparison for the MCAT data. (e) Selection curves for the OMCAT versus OWT comparison for the MCAT data. (f) Selection curves for the YWT versus OWT comparison for the MCAT data.

Figure 2 depicts the level of agreement between combined p and each of the other methods. Specifically, for a given number of top ranked genes by combined p we calculate the proportion that appear in the group of top ranked genes by each of the other methods. We examine this proportion over a range of values and call the resulting graphs selection curves. The selection curves for each of the three spike-in datasets and the three comparisons from the MCAT data are shown in Figure 2.

The selection curves show that the ANOVA and median t methods seem to agree well with combined p in most cases. This agreement is most likely due to the fact that these three testing methods are based on probe values instead of expression indices. The Affymetrix Latin Square data provides an exception – the median t method does not agree well with combined p for the initial group of genes selected. From the FDR and ROC plots we see that all methods are accurately detecting differentially spiked transcripts. Also, from the selection curves we see that the agreement between methods is good when the top 3000 genes are compared. This indicates that while the differentially spiked transcripts are being ranked high by all methods, the ranking within the group of differentially spiked transcripts varies by method. This is not surprising when we consider that the p-values for the top ranked probe sets are very small – less than 10^-20 ^for the 1000 top ranked genes from any method.

### Comparison of Methods using the MCAT qRT-PCR validated genes

A total of 47 genes from the MCAT study were validated using qRT-PCR. In Table 4 we show the proportion of qRT-PCR assayed genes ranked in the top 100, 150 and 200 genes for each method and each comparison. Note that we do not expect all 47 genes to be selected for any one comparison. The combined p method is consistent with the other accepted methods.

We also examined the Spearman correlation between p-values calculated using the Affymetrix array data with each of the six testing methods and qRT-PCR data for the 47 qRT-PCR assayed genes. We note that because the testing methods are based on information from the same subjects, we expect dependence among p-values and consistency in ranking for the most significant p-values. The 47 genes were not selected randomly and tend to have smaller p-values and higher rankings when compared to the full distribution. Spearman correlation captures the level of agreement of the rankings by the different methods. For the YMCAT vs YWT comparison, the correlation between p-values from the six testing methods applied to the array data is greater than 0.70 for any pair of methods, while the correlation between p-values from the array and qRT-PCR data is less than 0.45 for any of the testing methods. For the OMCAT vs OWT comparison, the correlation between p-values from the six testing methods applied to the array data is greater than 0.85 for any pair of methods, while the correlation between p-values from the array and qRT-PCR data is less than 0.30 for any of the testing methods. For the YWT vs OWT comparison, the correlation between p-values from the six testing methods applied to the array data is greater than 0.85 for any pair of methods, while the correlation between p-values from the array and qRT-PCR data is less than 0.20 for any of the testing methods. This shows that the testing methods (applied to the Affymetrix array data) are ranking the 47 qRT-PCR validated genes similarly. However, the correlation between p-values based on qRT-PCR data and Affymetrix array data are only weakly correlated.

### Combined P and Probe Level Tests as Diagnostics

Combined p-values and probe level p-values can be used as diagnostics. It is appropriate to apply the combined p method for both of the one-sided tests. For diagnostic purposes it is interesting to compare the two one-sided combined p-values for each probe set. If all probes consistently indicate a change in one direction (up- or down-regulation), then we would expect one of the combined p-values to be close to zero and the other to be close to one. However, if some probes indicate differential expression in opposing directions, it is possible to obtain small combined p-values in both directions. These probe sets can be easily identified by plotting the combined p-values against each other.

Probe level tests can also be used as a diagnostic. Specifically, we can examine the t-statistics for each probe of a probe set. The number of probes indicating up-regulation and down-regulation can be tabulated. For some probe sets, we might find probes indicating differential expression in opposing directions. For a given probe set, let *n*_*up *_be the number of probes exhibiting statistically significant evidence of up-regulation and *n*_*down *_be the number of probes exhibiting statistically significant evidence of down-regulation at a specified false positive rate. Then the level of discordance for the probe set can be summarized by *d *= *min*(*n*_*up*_, *n*_*down*_).

We illustrate the use of the combined p-value and probe level tests as diagnostics using the Golden Spike data. Recall that for the Golden Spike data there are 1331 probe sets that correspond to differentially spiked transcripts. All of the *log*_2_(*FC*) values for the differentially spiked transcripts are positive. The combined p-values (testing for both up- and down-regulation) for each probe set were calculated. Probe set 154940-at has small combined p-values in both directions (1.81 × 10^-6 ^and 1.24 × 10^-11^.)

We performed a t-test for each of the probes. Using a p-value cutoff of 0.05, the number of probes indicating up-regulation (*n*_*up*_) and down-regulation (*n*_*down*_) were tallied by probe set. The majority of probe sets have discordance values of zero (57%) or one (33%). Only 18 probe sets have discordance values of four or greater; three of these correspond to differentially spiked transcripts. Probe set 154940-at has a discordance value of five – the largest observed for this dataset.

Probe set 154940-at corresponds to a differentially spiked transcript with known *log*_2_(*FC*) = 1.32. The probe level t-statistics and rankings for this probe set are shown in Table 5. Five consecutive probes have significant t-statistics ranging from -2.30 to -17.72. Eight of the probes have significant positive t-statistics ranging from +2.15 to +15.11. The probe sequences for this probe set were obtained from Affymetrix and a nucleotide-nucleotide BLAST search against NCBI transcript reference sequences was performed. The eight probes with positive t-statistics mapped only to CG6876 (represented by probe set 154940-at). The five probes with significant negative t-statistics mapped to CG6876 and CG7011 (represented by probe set 152984-at). Probe set 152984-at was differentially spiked with *log*_2_(*FC*) = 1.81, so it is not clear why these probes would be exhibiting evidence of down regulation.

Probe level t-statistics can also be used to screen for outlying probes. A probe that is acting differently than other probes within the same probe set could be indicative of cross hybridization or alternative splicing. As an example, we consider probe set 146788-at for which the majority of the probes have t-statistics between -3.58 and +2.67, but one probe has a t-statistic of +48.25. The probe level t-statistics and rankings for this probe set are shown in Table 5. This probe set does not correspond to a differentially spiked transcript and the majority of probes seem to reflect this. However, a single probe seems to be showing strong evidence of differential expression. The sequence for this probe was obtained from Affymetrix and a nucleotide-nucleotide BLAST search was performed. A 15 bp match to the probe sequence was found. The matching sequence corresponds to CG5003 which is represented by probe set 154310-at on the DrosGenome1 array. Furthermore, for the Golden Spike experiment, this transcript was differentially spiked with known *log*_2_(*FC*) = 2. So, in this case, there is a plausible explanation for the behavior of the outlying probe.

### Illustration of differences between Median t and Combined P methods

The combined p and median t methods are both based on probe level tests of significance and seem to rank genes similarly in many cases. To examine the instances when the combined p and median t methods diverge, we consider probe sets 151862-at and 153401-at from the Golden Spike data. The t-statistics and rankings for these probe sets are shown in Table 5. For probe set 151862-at, the median t method ranks this probe set as 698 while the combined p method ranks it as 1173. Eight of the probe level tests have t-statistics ranging between 3.47 and 10.61. Hence more than half the probes indicate up-regulation of the corresponding gene. In contrast, for probe set 153041-at, the combined p method ranks this probe set as 603 while the median t method ranks it as 1822. Seven of the probe level test have t-statistics ranging from 3.08 to 29.19. Here half of the probes indicate up-regulation, some with very large t-statistics. This demonstrates that although the combined p and median t methods perform similarly, they weight evidence in different ways. Large t-statistics which are greater than the median have no effect on the median t, while the combined p method gives them higher weight.

## Discussion

There are two possible objectives of testing for differential gene expression using microarray data. One goal is to determine whether or not a particular gene is differentially expressed. Another goal is to rank the genes in order of evidence of differential expression. All methods considered here produce p-values which can be used for testing and ranking.

If we are interested in determining whether a particular gene is differentially expressed, then it is important to control the false positive rate. In practice, this is difficult. The observed false positive rates for each of the methods when a raw p-value cutoff of 0.01 is used to identify differentially expressed genes are shown in Table 3. None of the methods maintain a false positive rate close to the stated *α *level of 0.01. In addition to calculating the error rates based on raw p-values, we also examine the error rates based on a calibrated p-value. Specifically, our calibration set is comprised of those probe sets called Absent on all arrays according to the MAS Absent/Present call algorithm. Using a calibrated p-value reduces the false positive rates for all methods except median t. However, the only way to precisely control the false positive rate would be to calibrate using all or a randomly selected set of equally expressed genes. Of course, if we knew which genes were differentially expressed, we would not have to test for differential expression.

In practice, investigators are often more concerned with ranking genes in order of evidence of differential expression. For this objective, the correctness of the ranking is more important than maintaining the stated false positive rate. The ROC curves, FDR plots and rankings of the true positives illustrate the relative abilities of each of the methods to rank the true positives. While ROC curves allow us to examine the power over a range of false positive rates, they do not tell us what p-value cutoff to choose to achieve a desired false positive rate.

When reviewing the results from Tables 1 and 3, it is clear that methods do better at ranking genes rather than maintaining stated false positive rates. In addition, method performance based on false positive rate is highly dependent on the dataset. To remedy this, we believe that some type of data specific calibration is necessary. We have proposed one such calibration approach, but this is an area for further research. In contrast, ranking appears to be more consistent across methods and datasets. This explains why many authors look at ROC curves. We note that calibration of p-values using monotonic transformations will not affect rankings. So, while we may not know the correct threshold value for declaring significant differential expression, we are better able to identify top candidates for differential expression. We can see from Tables 1 and 3 that the relative performance based on false positive rate is distinct from relative performance based on ranking. Considering all of these factors, we place more emphasis on ranking genes in order of differential expression.

With the goal of ranking genes in order of differential expression in mind, the combined p and median t methods perform well for all three datasets considered. Based on the rankings of known true positives and power by intensity we see that the combined p method offers slightly improved power as compared to the median t method for the Golden Spike and Gene Logic Tonsil datasets. For the Affymetrix Latin Square data, Cyber-T offers improved power when a false discovery rate of 0.05 is desired.

The selection curves shown in Figure 2 show that gene rankings based on combined p are highly correlated with the rankings by ANOVA and median t. The one exception is seen in the selection curves for the Affymetrix Latin Square data, where combined p and ANOVA methods seem to be ranking very similarly to each other, but different from the other methods. However, all of the methods yield high rankings and very small p-values for the differentially spiked transcripts for this dataset. This implies that the ranking within the group of differentially spiked transcripts varies by method.

Our comparison employs spike-in datasets for which the truth is assumed to be known. We include results based on the Golden Spike data. Recently deficiencies of the Golden Spike data have been noted [22,23]. The most relevant issue for this study is that the null distribution of p-values (for transcripts known to be equally expressed) is not uniform. This problem is not unique to the Golden Spike Data. We have observed a non-uniform null distribution in the other spike-in datasets. In our experience, even "real" biological datasets can exhibit evidence of a non-uniform null distribution. Since we are only interested in the relative performance of the methods we feel it is appropriate to include the Golden Spike data in our comparison. We note that in the original Golden Spike comparison, a probe set level normalization was performed (in addition to a probe level normalization) because "many of the expression summary data sets that were produced still show a dependence of fold change on the signal intensity" [14]. We acknowledge that, in all likelihood, testing methods based on probe set summary values would have exceeded the performance of the methods based on probe level tests had a probe set level normalization been performed for the Golden Spike data. However the dependence of fold change on signal intensity seems to be an artifact of the Golden Spike data and not typical of other datasets [23]. The probe set normalization improved the performance of the methods based on ROC curves, but not necessarily estimated fold change. It would seem that if the combination of probe and probe set level normalizations really offered improved performance for a variety datasets, then both types of normalization would be implemented in commonly used algorithms. Instead, algorithms include either a probe level normalization [2,3] or probe set level normalization [1]. It should be clear that a probe set level normalization does not apply to methods based on probe level tests of significance.

We note that the median t method is a special case of the ChipStat algorithm. The ChipStat algorithm uses probe level comparisons to detect differential gene expression [9]. Specifically, PM-MM differences are used to perform individual probe level significance tests using the t-test. The number of probe pairs changing in a given direction, with p-values less than a fixed value (denoted *p*_*ps*_), is tabulated and used as a measure of the significance of change in gene expression. It is up to the user to choose both the value of *p*_*ps *_and the number of probe pairs required in order to declare a probe set differentially expressed. If the PM values, instead of the PM-MM differences, are used and at least half of the probes within a probe set must be statistically significant to declare the probe set differentially expressed, then this method reduces to the median t method.

The combined p method is particularly well suited as a diagnostic tool for exploratory analysis of microarray data. In particular, the two one-sided combined p-values can be used to screen for outlying probe sets. In addition, probe level t-statistics, upon which the combined p method is based, can be used to identify outlying probes within a probe set. Unusual probe sets can be flagged for further examination. In some cases, this type of behavior may be an indication of alternative splicing or cross hybridization. A discussion of methods for detecting alternative splicing using microarray technology is given by [24].

## Conclusion

The combined p method is a promising alternative to existing methods of testing for differential gene expression. The combined p and median t methods are both based on probe level tests of significance and perform well based on ranking genes in order of evidence of differential expression. One exception is the Affymetrix Latin Square data where the combined p and median t do not agree well for the rankings of the top 1000 genes. However, the main difference between the combined p and median t methods lies in how they weight evidence. Large t-statistics which are greater than the median have no effect on the median t, while the combined p gives them higher weight. The combined p method also leads to useful diagnostics. In particular, it allows us to examine conflicting information provided by probes within a probe set. A further examination of such conflicting information may point to outlying probes or lead to interesting discoveries. The median t on the other hand, makes its decision regarding differential expression based on the "median probe" and does not pay attention to any discordance that may be present among probes. This presumably makes the median t more robust at the expense of missing interesting phenomena, such as alternative splicing or cross hybridization.

## Methods

For all methods considered, the hypotheses may be stated as *H*_0 _: *μ*_*T *_= *μ*_*C *_versus *H*_*a *_: *μ*_*T *_≠ *μ*_*C *_where *μ*_*C *_is the expected *log*_2 _expression for some control group and *μ*_*T *_is the expected *log*_2 _expression for the treatment group. Depending on the method, the expression may be estimated using probe level expression or some computed probe set level expression index. Since *log*_2 _fold change is calculated as *log*_2_(*FC*) = *μ*_*T *_- *μ*_*C *_this is equivalent to testing *H*_0 _: *log*_2_(*FC*) = 0 versus *H*_*a *_: *log*_2_(*FC*) ≠ 0.

### Fisher's Combined P Method

Each PM probe in a given probe set can be used to estimate the relative transcript abundance for the gene corresponding to that probe set. One can also test for differential expression using each single probe separately. P-values from these individual probe level tests can be combined to provide an overall measure of evidence of differential expression.

Fisher (1932) proposed a method for combining p-values from independent tests of significance. For a fixed probe set, let *p*_*i *_be the p-value for the test using information from probe *i*, *i *= 1, ..., *m *and *s*_*i *_= -2*ln*(*p*_*i*_). Then under *H*_0_, *p*_*i *_~ *unif *(0, 1). Hence, si~χ22
 MathType@MTEF@5@5@+=feaafiart1ev1aaatCvAUfKttLearuWrP9MDH5MBPbIqV92AaeXatLxBI9gBaebbnrfifHhDYfgasaacH8akY=wiFfYdH8Gipec8Eeeu0xXdbba9frFj0=OqFfea0dXdd9vqai=hGuQ8kuc9pgc9s8qqaq=dirpe0xb9q8qiLsFr0=vr0=vr0dc8meaabaqaciaacaGaaeqabaqabeGadaaakeaacqWGZbWCdaWgaaWcbaGaemyAaKgabeaakiabc6ha+HGaciab=D8aJnaaDaaaleaacqaIYaGmaeaacqaIYaGmaaaaaa@34FF@ and ∑isi~χ2m2
 MathType@MTEF@5@5@+=feaafiart1ev1aaatCvAUfKttLearuWrP9MDH5MBPbIqV92AaeXatLxBI9gBaebbnrfifHhDYfgasaacH8akY=wiFfYdH8Gipec8Eeeu0xXdbba9frFj0=OqFfea0dXdd9vqai=hGuQ8kuc9pgc9s8qqaq=dirpe0xb9q8qiLsFr0=vr0=vr0dc8meaabaqaciaacaGaaeqabaqabeGadaaakeaadaaeqaqaaiabdohaZnaaBaaaleaacqWGPbqAaeqaaOGaeiOFa4hcciGae83Xdm2aa0baaSqaaiabikdaYiabd2gaTbqaaiabikdaYaaaaeaacqWGPbqAaeqaniabggHiLdaaaa@3995@. We reject *H*_0 _at the *α *level of significance if ∑isi>χ1−α,2m2
 MathType@MTEF@5@5@+=feaafiart1ev1aaatCvAUfKttLearuWrP9MDH5MBPbIqV92AaeXatLxBI9gBaebbnrfifHhDYfgasaacH8akY=wiFfYdH8Gipec8Eeeu0xXdbba9frFj0=OqFfea0dXdd9vqai=hGuQ8kuc9pgc9s8qqaq=dirpe0xb9q8qiLsFr0=vr0=vr0dc8meaabaqaciaacaGaaeqabaqabeGadaaakeaadaaeqaqaaiabdohaZnaaBaaaleaacqWGPbqAaeqaaOGaeyOpa4dcciGae83Xdm2aa0baaSqaaiabigdaXiabgkHiTiab=f7aHjabcYcaSiabikdaYiabd2gaTbqaaiabikdaYaaaaeaacqWGPbqAaeqaniabggHiLdaaaa@3D70@. A combined p method has previously been used to detect simultaneous matches to multiple patterns in sequence homology searches [25]. Here, we use the combined p method to detect differentially expressed genes using Affymetrix expression array data.

The combined p method can be used with any two-sample test, including the t-test and non-parametric tests such as the Wilcoxon rank sum test. For this paper, we use probe level t-tests. The probe level tests which form the basis of the combined p method should be one-sided tests. It doesn't make sense to combined significant p-values that indicate change in opposing directions. Of course, we are interested in a change in either direction (up- or down-regulation), so the combined p-value is calculated each of the one-sided tests. The minimum of the two one-sided combined p-values is used for all comparisons in this paper.

In the unusual situation where both p-values are below an established threshold value an explanation for the behavior should be sought. Hence the combined p method can be used as a diagnostic as well as a test. The individual probe level tests can also be used as a diagnostic. Some probe sets will contain probes that are providing conflicting information about the direction of the fold change. These probes can be flagged for further examination. In other cases, a probe set that represents a gene suspected of being differentially expressed may not be selected. In this case, the investigator can look at the probe level tests to understand why the probe set was not selected.

We considered using an adaptation of Fisher's combined p method that would allow for correlation between probes of the same probe set. An estimate of the correlation between probe level p-values for a probe set is required. If we assume an exchangeable correlation structure such that corr(*s*_*i*_, *s*_*i'*_) = *c *for *i *≠ *i'*, then the correlation can be estimated using a method of moments approach [26]. We considered the quadratic form

q=∑i=1m(si−s¯)2(m−1),
 MathType@MTEF@5@5@+=feaafiart1ev1aaatCvAUfKttLearuWrP9MDH5MBPbIqV92AaeXatLxBI9gBaebbnrfifHhDYfgasaacH8akY=wiFfYdH8Gipec8Eeeu0xXdbba9frFj0=OqFfea0dXdd9vqai=hGuQ8kuc9pgc9s8qqaq=dirpe0xb9q8qiLsFr0=vr0=vr0dc8meaabaqaciaacaGaaeqabaqabeGadaaakeaacqWGXbqCcqGH9aqpdaaeWbqaamaalaaabaGaeiikaGIaem4Cam3aaSbaaSqaaiabdMgaPbqabaGccqGHsislcuWGZbWCgaqeaiabcMcaPmaaCaaaleqabaGaeGOmaidaaaGcbaGaeiikaGIaemyBa0MaeyOeI0IaeGymaeJaeiykaKcaaaWcbaGaemyAaKMaeyypa0JaeGymaedabaGaemyBa0ganiabggHiLdGccqGGSaalaaa@444E@

which is the sample variance of the *s*_*i *_values. It can be shown that *E*(*q*) = 4(1 - *c*). Hence a method of moments estimate for *c *is *ĉ* = 1 - *q*/4. This leads to an approximate *χ*^2 ^distribution for ∑_*i*_*s*_*i*_. In practice, neither the estimated correlation nor the *χ*^2 ^approximation performed well. We found that the estimated correlation was extremely variable and that the performance of the method was weakened due to this variability. We note that it may not be appropriate to use the combined p method for tiling arrays in which there is typically considerable overlap between probes.

### Two-way ANOVA Method

A two-way ANOVA model can be fit to probe level intensity values for a given probe set [8]. For each probe set, the following model is imposed

*Y*_*ijk *_= *μ *+ *P*_*i *_+ *T*_*j *_+ *PT*_*ij *_+ *ε*_*ijk*_,

where *Y*_*ijk *_is the *log*_2_(*PM*) value corresponding to the *k*th replicate of treatment *j *for probe *i*, *μ *is the overall mean, *P*_*i *_is the effect of probe *i*, *T*_*j *_is the effect of treatment *j*, *PT*_*ij *_is the effect of the interaction between probe *i *and treatment *j*, and *ε*_*ijk *_is the error. To test for differential gene expression, a test of a treatment main effect is used.

### Cyber-T

Cyber-T is a regularized t-test based on expression indices [6]. This method was implemented using the *BayesReg *and *bayesAnova *R functions available from the Cyber-T website [27].

### Median t Method

The median t-statistic of the probes in a probe set can be used as a test statistic for differential expression of the whole probe set [10]. Specifically, t-statistics are calculated for each PM probe and the median t-statistic found among all PM probes in the probe set is found. When combined with a suggested normalization method involving the logit transformation, Lemon *et al. *called the resulting method the Logit-t. We focus only on the testing method, which we will refer to as median t.

### Original t Method

Here we use the Student's t-test applied to expression indices as a test of differential gene expression.

### Moderated t Method

This method is an empirical Bayes modification of the t-test [4]. This method is implemented through the *limma *package from Bioconductor [28].

## Abbreviations

FC: fold change

FDR: false discovery rate

FPR: false positive rate

IQR: inter-quartile range

MAS: (Affymetrix) Microarray Suite

MM: mis-match (probe)

PM: perfect match (probe)

qRT-PCR: quantitative reverse-transcription polymerase chain reaction

RMA: robust multi-array average

ROC: receiver operator characteristic

## Authors' contributions

AH participated in all phases of the method development, study design and analysis. HI assisted in the method development and study design. All authors read and approved the final manuscript.

## Figures and Tables

**Table 1:** IQR of Ranks for True Positives.

Data	Method	Q1	Median	Q3
Golden Spike	ANOVA	345.00	1624.00	3986.50
	Combined P	337.50	1173.00	3527.50
	Cyber-T	356.50	1490.00	4640.00
	Median t	339.50	1320.00	3719.00
	Moderated t	366.50	1543.00	5403.00
	Original t	445.50	1652.00	5545.50
	**Ideal**	**333.50**	**666.00**	**998.50**

Gene Logic Tonsil	ANOVA	182.25	363.50	1455.50
	Combined P	182.25	363.50	1031.00
	Cyber-T	182.25	363.50	3749.00
	Median t	182.25	363.50	2638.50
	Moderated t	182.25	365.50	27707.00
	Original t	182.25	390.50	39284.00
	**Ideal**	**182.25**	**363.50**	**544.75**

Affymetrix Latin Square	ANOVA	956.25	1951.50	3023.75
	Combined P	956.25	1946.50	2984.50
	Cyber-T	956.25	1957.50	2995.75
	Median t	956.25	1949.50	2998.75
	Moderated t	965.25	1961.50	3002.75
	Original t	965.25	1961.50	3002.75
	**Ideal**	**956.25**	**1911.50**	**2866.75**

This table shows the interquartile range (IQR) of ranks by method for true positives for each of the datasets. For the Golden Spike data there are a total of 1331 true positives. For the Gene Logic Tonsil data there are 66 comparisons on 11 differentially spiked transcripts for a total of 726 true positives. For the Affymetrix Latin Square data there are 91 comparisons on 42 differentially spiked transcripts for a total of 3822 true positives.

**Table 2:** Power by Intensity Range.

		Power by Intensity Quartile				
Data	Method	Q1	Q2	Q3	Q4	Overall Power
Golden Spike	ANOVA	0.096	0.192	0.295	0.465	0.262
	Combined P	0.144	0.315	0.413	0.568	0.360
	Cyber-T	0.042	0.141	0.259	0.477	0.230
	Median t	0.141	0.306	0.404	0.556	0.352
	Moderated t	0.060	0.159	0.265	0.396	0.220
	Original t	0.006	0.009	0.021	0.033	0.017

Gene Logic Tonsil	ANOVA	0.341	0.619	0.829	0.577	0.591
	Combined P	0.390	0.646	0.867	0.615	0.629
	Cyber-T	0.374	0.624	0.790	0.566	0.588
	Median t	0.368	0.630	0.851	0.599	0.612
	Moderated t	0.258	0.597	0.746	0.549	0.537
	Original t	0.203	0.547	0.713	0.473	0.483

Affymetrix Latin Square	ANOVA	0.418	0.819	0.915	0.813	0.741
	Combined P	0.518	0.887	0.945	0.895	0.811
	Cyber-T	0.646	0.925	0.986	0.972	0.882
	Median t	0.591	0.916	0.982	0.967	0.864
	Moderated t	0.584	0.888	0.968	0.927	0.841
	Original t	0.518	0.847	0.941	0.872	0.795

This table shows the power for each of the four intensity quartiles as well as the overall power for each of the datasets and methods. The power is calculated as the proportion of true positives that were detected while maintaining an overall false discovery rate of 0.05 or less.

**Table 3:** False Positive Rates.

Method	Golden Spike	Gene Logic Tonsil	Affymetrix Latin Square
ANOVA	0.286 (0.135)	0.146 (0.007)	0.096 (0.018)
Combined P	0.374 (0.110)	0.146 (0.003)	0.155 (0.007)
Cyber-T	0.190 (0.096)	0.037 (0.005)	0.047 (0.017)
Median t	0.004 (0.131)	2.8 × 10^-4 ^(0.008)	2.8 × 10^-4 ^(0.023)
Moderated t	0.160 (0.079)	0.027 (0.006)	0.034 (0.014)
Original t	0.123 (0.051)	0.024 (0.007)	0.035 (0.016)

This table shows the observed false positive rate calculated using a p-value cutoff of 0.01. The false positive rate based on a calibrated p-value of 0.01 is shown in parentheses. The calibrated p-value was calculated using probe sets called Absent on all arrays for a given experiment. For the combined p method, a p-value cutoff of 0.005 was used because we use the minimum of the two one-sided combined p-values.

**Table 4:** Proportion of the MCAT qRT-PCR assayed genes selected by method.

	YMCAT vs YWT			OMCAT vs OWT			OWT vs YWT		
Method	Top 100	Top 150	Top 200	Top 100	Top 150	Top 200	Top 100	Top 150	Top 200

ANOVA	0.277	0.362	0.489	0.085	0.106	0.106	0.128	0.128	0.128
Combined P	0.277	0.340	0.426	0.064	0.106	0.106	0.085	0.128	0.128
Cyber-T	0.213	0.255	0.362	0.064	0.106	0.149	0.149	0.149	0.149
Median t	0.255	0.362	0.426	0.043	0.064	0.085	0.106	0.128	0.128
Moderated t	0.085	0.106	0.128	0.021	0.043	0.043	0.128	0.128	0.128
Original t	0.021	0.043	0.064	0.000	0.000	0.000	0.064	0.085	0.106

This table shows the proportion of the 47 qRT-PCR assayed genes ranked in the top 100, 150 and 200 by each of the testing methods applied to the corresponding array data.

**Table 5:** Golden Spike Probe Set Examples.

						Rank by Method					
Probe Set	Ordered t-statistics					ANOVA	Combined P	Cyber-T	Median t	Moderated t	Original t

154940-at	-17.72	-13.75	-11.15	-5.34	-2.30	3513	1292	8596	1172	8515	7815
*log*_2_(*FC*) = 1.32	-0.38	2.15	4.14	4.68	7.2						
	8.34	9.16	12.57	15.11							

146788-at	-3.58	-2.44	-2.43	-2.22	-1.31	8410	6012	8172	8337	8679	7217
*log*_2_(*FC*) = 0	-1.06	-0.82	-0.11	0.14	0.44						
	1.14	1.44	2.67	48.25							

151862-at	-0.79	-0.21	-0.20	-0.12	0.03	2680	1173	2556	698	4030	5631
*log*_2_(*FC*) = 1.58	0.15	3.47	4.63	5.74	7.02						
	7.89	7.98	9.08	10.61							

153041-at	-0.48	-0.32	0.66	0.76	0.87	1664	603	1155	1822	1815	2968
*log*_2_(*FC*) = 1.81	1.37	1.69	3.08	3.42	10.25						
	18.85	19.24	19.57	29.19							

This table shows the ordered probe level t-statistics and rankings by method for selected probe sets from the Golden Spike data.
